# Selecting the optimal keyhole approach for internal carotid and middle cerebral artery aneurysms. Anatomical comparison of transorbital, lateral supraorbital and minipterional routes with clinical implications

**DOI:** 10.1007/s00701-026-06871-x

**Published:** 2026-04-13

**Authors:** Alejandra Mosteiro, Marta Codes, Gloria Cabrera, Xavier Montero Mena, Thomaz Topczewski, Hugo Andrade-Barazarte, Ivan Radovanovic, Alberto Prats-Galino, Alberto Di Somma, Joaquim Enseñat, Ramon Torné

**Affiliations:** 1 Departament de Cirurgia I Especialitats Medicoquirúrgiques, Universitat de Barcelona (UB), Facultat de Medicina I Ciències de La Salut, Barcelona, Spain; 2 Institut Clínic de Neurociències (ICN), Department of Neurological Surgery, Hospital Clínic de Barcelona, Barcelona, Spain; 3 Faculty of Physics, University of Barcelona, Barcelona, Spain; 4 Division of Neurosurgery, Department of Surgery, Toronto Western Hospital, University Health Network, Toronto, ON Canada; 5 Department of Surgery, University of Toronto, Toronto, ON Canada; 6 Institut d’Investigacions Biomèdiques August Pi I Sunyer (IDIBAPS), Barcelona, Spain

**Keywords:** Transorbital, Minipterional, Lateral Supraorbital, Aneurysms, Surgical freedom

## Abstract

**Purpose:**

Among the keyhole approaches for the treatment of anterior circulation aneurysms, the minipterional (MPT) and lateral supraorbital (LSO) mini-craniotomies have gained great acceptance. With the recent introduction of the transorbital (TO) route to the Silvian fissure and carotid cistern, an objective analysis is required to stablish general indications and limits to each approach.

**Methods:**

Four heads (8 sides) were used for each of the three approaches (TO, LSO, MPT). All specimens underwent a basal CT scan for neuronavigation. Procedures were performed simulating a real surgical scenario, exposing four vascular targets: posterior communicating artery (PCom), internal carotid bifurcation (ICA), M1 segment and bifurcation of the middle cerebral artery (MCA). Surgical freedom (SF) and working angles were calculated at the four vascular targets, and mean differences were compared with Kruskal–Wallis. Transferability of data are demonstrated with three illustrative cases.

**Results:**

The ICA segments C6-C7, PCom and M1 segment and MCA bifurcation could be exposed ipsilaterally in all specimens and for all three approaches. With the PCom as a target, mean area of SF achieved was 36.19 (27.66) cm^2^ for TO, 71.35 (21.04)cm^2^ for LSO, and 101.59 (42.34)cm^2^ for MPT (p = 0.013). At the ICA bifurcation, the LSO offered the maximal SF area (85.34 IQR38.42 cm^2^) (p = 0.025), with ventral and horizontal angles also favouring the LSO. At the M1, there were no significant differences between the approaches. Notably, the TO approach provides its maximal SF and degree of instrument angulation at the M1 (68.02 IQR35.04 cm^2^). Manoeuvrability at the MCA bifurcation was maximal with the MTP (90.99 IQR26.88 cm^2^), although the differences did not reach statistical significance compared to LSO (72.02 IQR19.13 cm^2^) and TO (42.38 IQR3.37 cm^2^) (p = 0.132). Three clinical cases of unruptured MCA and ICA aneurysms are detailed.

**Conclusions:**

Our data supports the addition of a new ventral route – the TO approach – to the already available keyhole routed to the anterior circulation (MTP and LSO). Manoeuvrability analysis contemplates independently four major vascular targets, providing more detailed data to help select the most appropriate approach for each individual case.

**Supplementary Information:**

The online version contains supplementary material available at 10.1007/s00701-026-06871-x.

## Introduction

Since the introduction of the keyhole concept by Perneczky [13], the idea of operating with a decreasing trauma to the brain and adjacent tissues while maintaining or increasing the treatment efficiency has been constantly evolving [5, 18]. In this process, the minipterional (MPT) and lateral supraorbital (LSO) keyhole craniotomies have achieved great acceptance and become the preferred choice over the traditional pterional craniotomy in the elective treatment of anterior circulation aneurysms in most centres [4, 19]. Recently, the introduction of the transorbital (TO) approach to the cerebrovascular armamentarium has open a new door to the Silvian fissure and carotid cistern [1, 6, 14]. The TO seems to provide a direct ventral access to the first segment (M1) of the middle cerebral artery (MCA) [1, 22], complimentary to the lateral and ventrolateral perspectives already offered by the MPT and the LSO, respectively [4, 14].

Even when the choice of the preferred approach is largely based on surgeon experience, an objective analysis is required to stablish general indications and limits to each approach. Such comparison between the MPT and the LSO routes to access the MCA has already been offered by other groups, both based on anatomical and clinical observations [10, 21]. With the present anatomical study, we have extended the comparison to include the internal carotid (ICA) and the posterior communicating artery (PCom) as targets, and to contemplate the addition of the TO route to the available selection of keyhole craniotomies for elective aneurysm clipping.

## Methods

### Anatomical dissections

Ethics approval was obtained from the IRB of the University of Barcelona. All the dissections were performed at the Laboratory of Surgical Neuroanatomy at the University of Barcelona. Cadaveric head specimens were fixated with Cambridge solution. Four heads (8 sides) were used for each approach. A 4-mm-diameter rigid endoscope 18 cm long, with 0° optics (Karl Storz), connected to a light source through a fiberoptic cable (300 W Xenon; Karl Storz). A high definition 4 k camera was used for image adquisition (Endovision Telecam SL; Karl Storz). Additionally, a M500 surgical microscope (Leica Microsystems) was used.

Specimens underwent a basal multislice helical CT scan (Siemens Somaton GoTop software, VA30ASP03) using axial spiral slices of 0.5-mm thickness with a 0° gantry angle. Six screws were implanted in the vertex area and used as fiducials for neuronavigation (Brainlab).

**Fig. 1:**
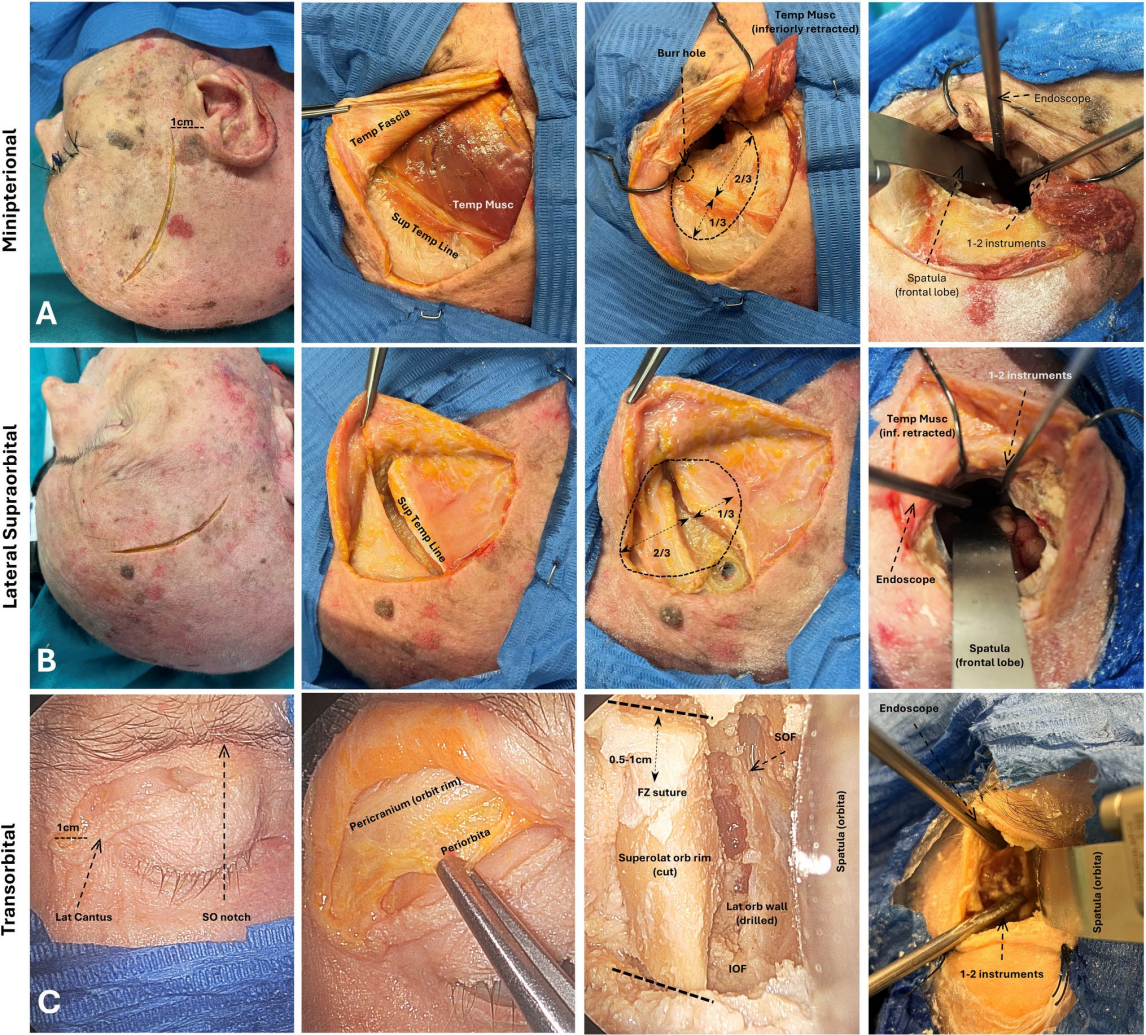
Initial steps for each approach: skin incision, craniotomy, and endoscopic-assisted instrument distribution. A) Minipterional approach. B) Lateral supraorbital approach. C) Transorbital approach with transitory lateral orbital rim removal

Procedures were performed simulating a real surgical scenario by four of the authors (AM, GC, MC, ADS). According to the preference and to the stage of the approach, either a microscopic or an endoscopic view was used. During the latter, either a 4-hand or a 2-hand technique was used, with the primary surgeon normally using 2 instruments and the secondary surgeon holding the scope and a complementary instrument, or an instrument and the suction if the endoscope was placed in an articulated holding arm (Fig. 1).

### Transorbital approach

The transorbital approach to the Sylvian and carotid cisterns was performed under a combined endoscopic and microscopic view. The orbital craniotomy included the transitory removal of the superolateral orbital rim. When targeting medially located structures (PCom), the meningo-orbital band (MOB) was cut to expose the ACP and an anterior clinoidectomy was performed extradurally (Fig. 1). The steps have been previously described elsewhere [8, 22].

### Minipterional approach

The standardised technique for the MPT can be found elsewhere [11] and is illustrated in Fig. 1.

### Lateral supraorbital approach

A detailed description of the LSO approach should be sought elsewhere [2] and is illustrated in Fig. 1. With this approach, the Sylvian fissure lies under the dural edge, below the sphenoid ridge (see below), and it can be seen by applying gentle pressure on the exposed frontal lobe.

### Remarks on opening of the silvian fissure

Arachnoid dissection to open the Sylvian cistern is performed differently in each of the approaches. In the TO route, the temporal pole along with the posterior region of the basal surface of the frontal lobe are exposed. Opening of the arachnoid between the two lobes leads directly to the sphenoidal compartment of the deep part of the Silvian fissure (i.e., the Sylvian cistern). Concretely, a separation is made between the temporal pole and subsequent temporal planum polare (inferiorly) and the posterior orbital gyri of the frontal lobe. As the M1 segment is reached, the dissection can be continued laterally (proximal to distal direction) towards the operculo-insular compartment of the Sylvian fissure, finally exposing the MCA bifurcation. Alternatively, the dissection can be continued medially (distal to proximal direction), to open the Sylvian vallecula and communicating the sphenoidal compartment of the Sylvian cistern with the carotid and optico-carotid cisterns. During these manoeuvres, dynamic retraction is applied mainly to the frontal lobe, whilst temporal retraction is limited to the temporal pole.

In the MPT approach the Sylvian fissure lies in the middle of the surgical field. Again, opening of the fissure can be performed from proximal to distal or vice versa, as explained for the LSO approach. Yet, the MPT allows for either frontal or temporal lobe retraction, which may ease the dissection in certain types of Sylvian fissure configurations [23].

In the LSO route, the Sylvian fissure lies at the inferior limit of the craniotomy. The dissection of the fissure can be performed from proximal to distal, starting at the optico-carotid region, or from distal to proximal, starting at the anterior Sylvian point. In this approach, the dissection is mainly performed by gentle distraction of the frontal lobe, as the temporal lobe is not accessible.

Following arachnoid dissection, ICA, MCA, ACA, and PCom were exposed in all three approaches, and stereotactic measurements for the each segment and each approach were obtained.

### Quantitative anatomical analysis

A virtual 3D model was created using Brainlab Neuronavigation. Surgical manoeuvrability and working angles were calculated at four specific vascular targets. The PCom target was selected at the point in which the PCom emerged from the ICA. The ICA bifurcation target was selected at the point where the ICA splitted into the anterior cerebral artery (ACA) and the MCA. The M1 target was selected at the midpoint of the M1 segment, defined as the midpoint between the ICA bifurcation and the MCA bifurcation. The MCA bifurcation target was selected at the point in which the MCA bi/trifurcated.

The surgical freedom (instrument movement) was calculated circumferentially in the surface of each approach. This was done by capturing point by point – with the Brainlab navigation wand – the maximal manoeuvrability attainable with a straight 25-cm instrument placed at each target and angulated circumferentially in the surface of each approach. A pyramid reconstruction was obtained with an Open Access software for quantitative data retrieval. The apex is placed at the surgical target, and the base corresponds to the captured points in the surface of the approach. The area at the base of the pyramid was designated as the surgical freedom and expressed in mm^2^. Based on these measurements, the maximal (vertical) and axial (horizontal) angles achievable with each approach were calculated for each target.

Differences in the abovementioned parameters were compared by means of Kruskal–Wallis. A p value of 0.05 was set for statistical significance. All calculations were done with SPSS Statistics v30.0 (IBM Corp.).

### Illustrative cases

The results obtained in the laboratory were transferred to the operating room, with an illustrative case for each approach herewith provided.

## Results

**Fig. 2:**
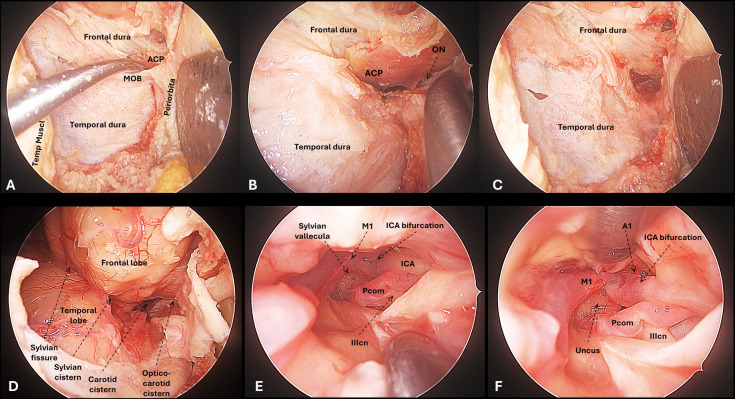
Targeting the PCom and ICA bifurcation through the transorbital approach. A) After lateral orbital craniotomy, the dura overlying the temporal and frontal lobes is exposed. The periorbita is gently retracted medially and the meningo-orbital (MOB) band is recognised. B) The MOB is cut to expose the anterior clinoid process (ACP) and the optic nerve (ON) exiting the optic canal. C) An anterior clinoidectomy is performed to increase the surgical field of view in the medial aspect of the approach when targeting these mesial vascular structures. D) The dura is opened over the Sylvian fissure, and the frontal and temporal lobes are exposed. The Sylvian fissure split starts at a midpoint between proximal (carotid cistern) and distal (Sylvian point). This gives access to the Sylvian cistern, where the M1 segment is located. The dissection is continued proximally towards the carotid and optico-carotid cisterns. E–F) The PCom and ICA bifurcation are seen from the TO perspective. The A1 segment lies deep within the field, and its exposure would require substantial frontal lobe retraction

**Fig. 3:**
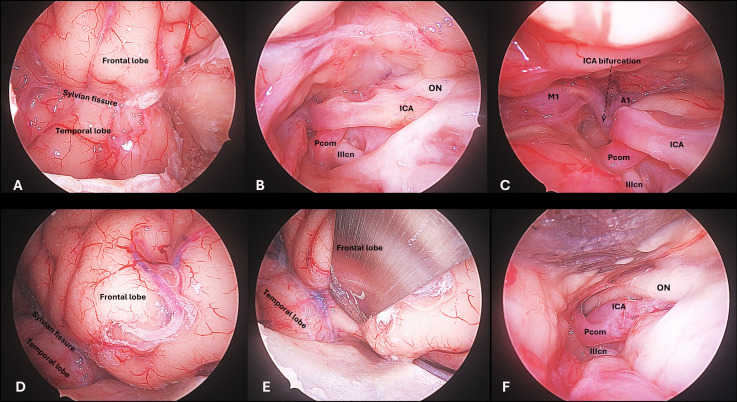
Targeting the PCom and ICA bifurcation through the minipterional (A-C) and lateral supraorbital (D-F) approach. A) After a MPT craniotomy, the frontal and temporal opercula are exposed, with the lateral sylvian fissure in between. B) Sylvian fissure is opened proximally to expose the optico-carotid cisterns, where the ICA and PCom are recognised. C) As the dissection continues towards the carotid cistern and the Sylvian vallecula, the ICA bifurcation along with the M1 segment are exposed. D) After a LSO craniotomy, the frontal operculum is exposed, with the Sylvian fissure lying at the inferior limit of the craniotomy and the temporal lobe hardly accessible. E) Sylvian fissure is opened by gentle separation of the frontal lobe. F) Proximal opening reveals the optico-carotid cisterns, where the ICA and PCom are recognised

**Fig. 4:**
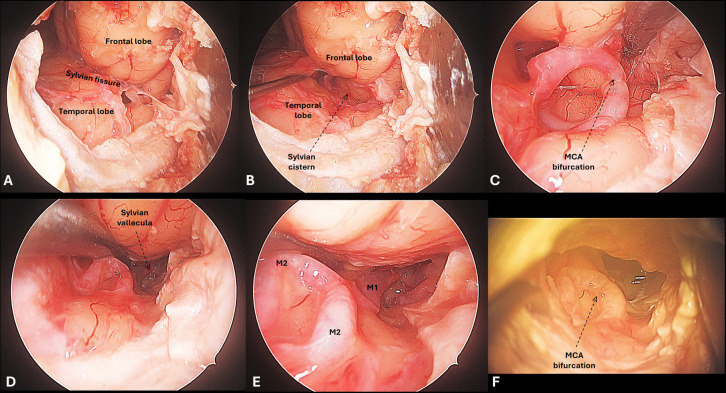
Targeting the MCA through the transorbital approach. A) After the dura is opened over the Sylvian fissure, the frontal and temporal lobes are exposed. B) The Sylvian fissure is opened by gentle retraction of the temporal pole. C) This gives access to the Sylvian cistern, where the M1 and/or the MCA bifurcation are located, depending on the length of the M1 segment. D-E) The Sylvian cistern can be opened more proximally to expose the proximal M1 segment. F) The MCA bifurcation as seen under the microscope through the TO approach

**Fig. 5:**
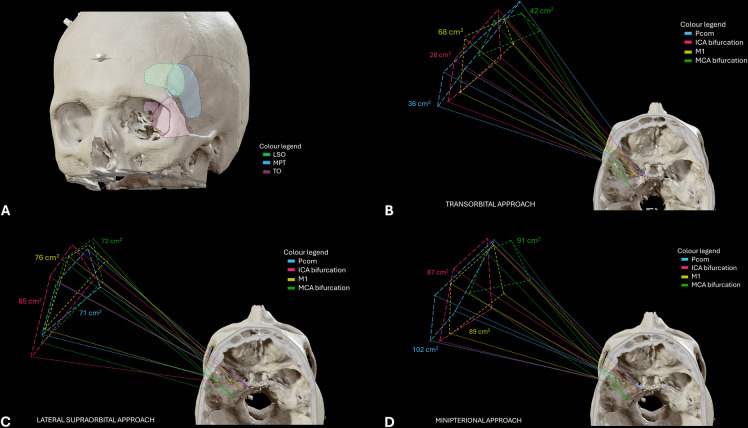
Surgical freedom with the three minimally invasive approaches. A) Schematic representation of the three craniotomies: Lateral supraorbital (LSO) in green, minipterional (MPT) in blue, and transorbital (TO) in purple. B-D) Three-dimensional reconstruction of the skull base, where the four vascular targets have been plotted according to neuronavigation measurements. A geometric pyramid-like reconstruction has been made to illustrate the surgical freedom area achieved for each vascular target, namely PCom (blue), ICA bifurcation (pink), M1 (yellow), and MCA bifurcation (green)

Illustrations of the intradural view with the TO, LSO and MPT approaches can be seen in Figs. 2, 3 and 4. ICA segments C6-C7, PCom, M1 segment and MCA bifurcation could be exposed ipsilaterally in all specimens and for all three approaches. Surgical manoeuvrability-related measurements are provided below and summarized in Fig. 5, in Table 1, and in Supplementary Tables S1-S4.

**Table 1:** Quantitative anatomical comparison of the transorbital (TO), lateral supraorbital (LSO) and minipterional (MTP) approaches to the posterior communicating (PCom), the internal carotid (ICA) and the middle cerebral (MCA) arteries. Data are given as median (IQR)

Vascular target	TO	LSO	MPT	p value
**PCom**				
Surgical freedom area (cm^2^)	36.19 (27.66)	71.35 (21.04)	101.59 (42.34)	**0.013**
Ventral angulation (º)	21.66 (5.76)	30.15 (1.11)	25.93 (16.02)	0.094
Horizontal angulation (º)	21.69 (7.27)	29.75 (2.03)	42.44 (8.90)	**0.013**
**ICA Bifurcation**				
Surgical freedom area (cm^2^)	28.77 (40.87)	85.34 (38.42)	86.76 (23.03)	**0.025**
Ventral angulation (º)	20.73 (5.23)	36.88 (8.61)	34.15 (10.91)	**0.016**
Horizontal angulation (º)	22.58 (9.18)	29.35 (6.38)	31.36 (1.42)	0.103
**M1 segment**				
Surgical freedom area (cm^2^)	68.02 (35.04)	76.48 (11.65)	88.86 (5.79)	0.276
Ventral angulation (º)	25.86 (42.58)	35.06 (3.78)	38.54 (17.28)	0.626
Horizontal angulation (º)	25.87 (29.91)	27.52 (4.03)	29.72 (4.10)	0.726
**MCA Bifurcation**				
Surgical freedom area (cm^2^)	42.38 (3.37)	72.02 (19.13)	90.99 (26.88)	0.132
Ventral angulation (º)	19.40 (5.05)	32.93 (8.92)	40.83 (4.02)	**0.020**
Horizontal angulation (º)	23.90 (20.41)	31.17 (9.81)	28.53 (15.33)	0.761

### Posterior communicating artery

PCom was accessible with all three approaches. With the TO route, the PCom and in some cases the ICA bifurcation lies medially to the medial limit of the corridor; for these cases, an anterior clinoidectomy widens the surgical field of view, further an extension of the craniotomy laterally towards the pterion offers a wider lateral angulation of the instruments, which favours the view of these mesial structures (Fig. 2). With the MPT route, the PCom is readily accessible after drilling of the lesser sphenoid wing and opening the optico-carotid and crural cisterns (Fig. 3A-C). With the LSO route, the PCom represents the closest (more superficial) and most accessible target after a proximal opening of the cisterns (Fig. 3D-E).

Taking the PCom as a target, the mean area of SF was 36.19 (IQR 27.66) cm^2^ for TO craniotomy, 71.35 (IQR 21.04) cm^2^ for LSO, and 101.59 (IQR 42.34) cm^2^ for MPT (95% CI 36.66–105.62, p = 0.013). The SF when targeting the PCom was significantly superior with the MPT compared to the TO (mean difference 93.40 cm^2^, 95% CI 58.13–16.60, p = 0.030). However, no significant differences were seen between the MTP and the LSO, or between the LSO and the TO for this target (Fig. 5 & Table S1).

The degree of ventral angulation when targeting the PCom was similar for the three keyhole approaches (95% CI 19.82–35.29, p = 0.094). Meanwhile, the degree of horizontal angulation was significantly superior with the MPT compared to both the LSO and the TO (95% CI 23.84–34.72, p = 0.013) (Table 1 & S1).

### Internal carotid artery bifurcation

The ICA bifurcation was accessible with all three approaches. With the TO route, the ICA bifurcation is accessed by distal to proximal Slvian fissure opening and communication with the carotid cistern. This target lies deep withing the TO surgical field, however, it is reachable after adequate arachnoid dissection; meanwhile, exposure of the A1 segment would require substantial frontal lobe retraction (Fig. 2). The ICA bifurcation is readily accessed with the MPT and LSO by proximal Sylvian fissure opening (Fig. 3).

At the ICA bifurcation, the MPT craniotomy offered the maximal SF (86.76 IQR 23.03 cm^2^), similar to the LSO (85.34 IQR 38.42 cm^2^) and statistically superior compared to the more restricted SF area achieved with the TO (28.77 IQR 40.87 cm^2^) (95% CI 42.75–88.07, p = 0.025) (Figs. 5). Ventral and horizontal degrees of angulation also favoured the LSO over the MTP and particularly over the TO at the ICA bifurcation (Table 1 & S2).

### Horizontal (M1) segment of the middle cerebral artery

The M1 segment was accessible with all three approaches. With the TO route, this was the most favourable surgical target, as it was the most superficially located and it lied right in the middle of the surgical field (Fig. 5). With the MPT and LSO routes, the M1 segment could be reached either by proximal to distal or distal to proximal Sylvian fissure split. With the LSO, the distal M1 lied in the most lateral margin of the approach in cases of a long M1 segment, which subjectively limited the dissection at this level.

At the M1, there were no statistically significant differences in any of the manoeuvrability measurements between the three keyhole approaches. Indeed, the maximal manoeuvrability in all approaches was achieved at this vascular segment (Table 1 & S3). Particularly, the TO approach seems to provide its maximal surgical freedom (68.02 IQR 35.04 cm^2^), and its maximal ventral (25.86 IQR 42.58º) and horizontal (25.87 IQR 29.91º) angulations at the M1 segment (Fig. 5) (Table 1 & S3).

### Middle cerebral artery bifurcation

The MCA bifurcation could be accessed with all three approaches. The comfortability to access this most lateral vascular target varied according to the interindividual differences in the length of the M1 segment when using ventral (TO) and ventrolateral (LSO) approaches, but not when using the lateral (MPT) approach (Fig. 4). In fact, with the MPT approach the MCA bifurcation was the most accessible target, as being superficially located with the option of direct access with a focused distal opening of the Sylvian fissure.

It should be noted that the perspective offered by the three approaches differed significantly. Although this stands for all vascular segments, it may be more important at the MCA bifurcation given the variable projection of the M2 branches.

Manoeuvrability at the MCA bifurcation was maximal with the MTP craniotomy (90.99 IQR 26.88 cm^2^), although differences did not reach statistical significance compared to the LSO (72.02 IQR 19.13 cm^2^) and the TO (42.38 IQR 3.37 cm^2^) (95% CI 59.57–10.39, p = 0.132) (Figs. 5). Ventral angulation favoured MTP over the other approaches (95% CI 26.75–49.64, p = 0.020), while horizontal angulation seemed similar among the three approaches (95% CI 21.48–44.32, p = 0.786) (Table 1 & S4).

### Illustrative cases

**Fig. 6:**
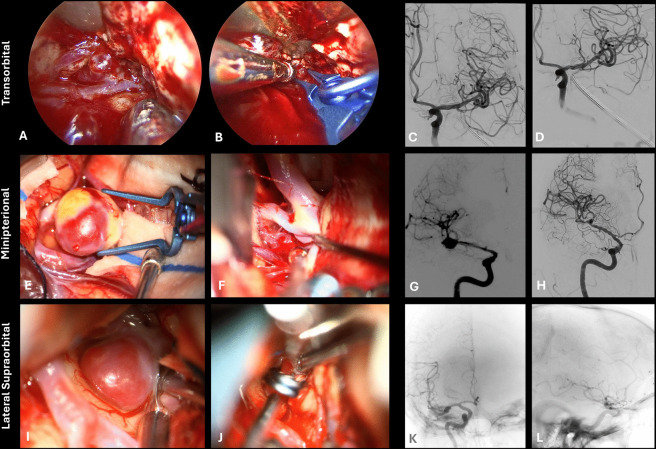
Illustrative surgical cases of clipping unruptured aneurysms through keyhole approaches. The transorbital (A-D) route was chosen for a laterally projecting MCA bifurcation aneurysm. Opening of the Sylvian cistern at the level of the temporal pole by gentle frontal retraction directly exposed the MCA bifurcation and the aneurysm (A). A clip can be placed comfortably though this route (B). Preoperative (C) and intraoperative (D) angiograms were obtained in the setting of a hybrid operating room. The minipterional (E–H) approach was chosen for a case of double aneurysm clipping, including an anteroinferior projecting MCA bifurcation aneurysm (E) and an inferiorly projecting ICA bifurcation aneurysm (F). Preoperative (G) and intraoperative (H) angiograms were obtained in the setting of a hybrid operating room. The lateral supraorbital (I-L) approach was selected for a posterolateral projecting MCA bifurcation aneurysm in a case with a short M1 segment. Targeted opening the superficial Sylvian fissure revealed the MCA bifurcation and aneurysm (I) and a clip was comfortably placed (J). Preoperative (K) and intraoperative (L) angiograms were obtained in the setting of a hybrid operating room

Figure 6 provides three illustrative cases where the keyhole approaches were used for the clipping of unruptured intracranial aneurysms of the MCA and ICA.

Table 2 summarises the aneurysm locations and projections favourable for transorbital clipping according to anatomical data.

**Table 2:** Descriptive summary of the aneurysm locations and projections favourable for transorbital clipping according to anatomical data

Arterial segment	Aneurysm projection/specific location		
	**Favourable**	**Intermediate**	**Unfavourable**
**Paraclinoid**	Clinoidal (lateral)	Ventral	Carotid cave
	Ophthalmic		Superior hypophyseal
	Dorsum		
**Posterior communicating**	Supratentorial		
	Infratentorial		
**Carotid bifurcation**		Anterior	Posterior
**A1**			Any
**M1**	Any		
	Anterior temporal		
**M2**	Short M1	Long M1	

## Discussion

An objective anatomical comparison between three keyhole approaches to the Sylvian and carotid cisterns is hereby provided. Major contributions of this work to the existing literature are the addition of a new ventral route – the transorbital approach – to the already available comparison between minipterional and lateral supraorbital craniotomies. Moreover, the surgical manoeuvrability analysis contemplates independently four major surgical targets to the anterior circulation, thus providing more detailed data to help select the most appropriate approach for each individual case.

### Transorbital approach

The TO approach represents the most ventral route to the anterior circulation among the three keyhole craniotomies analysed. With the TO, removal of the lesser sphenoid wing provides exposure of the Sylvian fissure from a ventral perspective, as demonstrated here and by others [1, 6, 14, 22]. Clinoidectomy is optional and it further exposes the mesial structures, mainly the optico-carotid cistern and the C5-C6 segments of the ICA(9). In the present study, it was only deemed necessary to enlarge the surgical field when accessing the Pcom. Transitory removal of the superolateral orbital rim was performed in all cases, as it diminished the need for orbital content retraction and it facilitated the use of multiple instruments and a spatula if needed. An optional extension of the orbital craniectomy to MacCarty’s point improves the visualization of the optico-carotid region [25] and it may be considered to increase the lateral angulation of the instruments when accessing the PCom.

A particularity during the TO approach is Sylvian fissure split. Given its ventral perspective, the TO route exposes the temporal pole and basal surface of the frontal lobe, thus arachnoid dissection opens to the sphenoidal compartment of the deep Silvian fissure (i.e., Sylvian cistern) [27]. Dissection leads directly towards the M1 segment. It may then be continued laterally (proximal-to-distal direction) towards the operculo-insular compartment of the Sylvian fissure exposing the MCA bifurcation, or medially (distal-to-proximal), towards the Sylvian vallecula and carotid cisterns. During these manoeuvres, dynamic retraction is applied mainly to the frontal lobe, while temporal retraction is mainly restricted to the temporal pole. Although the same principles for arachnoid dissection apply, the anatomical references and the bidirectional perspective gained with the TO route significantly differ from the conventional Sylvian split performed through the MPT and LSO [10, 27].

### Lateral supraorbital approach

LSO approach was proposed as an evolution of the keyhole supraorbital craniotomy by Hernesniemi [12]. In the LSO, opening of the temporal muscle is limited to its anterior portion, which reduces the risk of temporomandibular joint problems and muscle atrophy. Also, the frontal branch of the facial nerve remains unexposed. Access to the Willis polygonum is achieved through a subfrontal trajectory and dissection of the proximal Sylvian fissure [12].

The most accepted indication for the LSO is the treatment of anterior fossa lesions, particularly Acom aneurysms in cerebrovascular field. However, this antero-lateral route has also been proposed for more laterally located lesions, such as MCA aneurysms [26]. A major obstacle for the intradural exposure is the sphenoid ridge, hampering access to the temporal opercula and temporomesial structures. To overcome this restriction a modification known as “extended LSO” has been proposed, with the inner drilling of the sphenoid ridge providing a complete exposure of the Sylvian fissure and access to lesions within the mesial temporal region, ultimately achieving a similar surgical field and projection as with the pterional craniotomy [3].

### Minipterional approach

MTP craniotomy was introduced by Figueiredo et al. as an alternative to conventional pterional craniotomy for unruptured MCA aneurysms [11]. It has now become the preferred approach for MCA aneurysms in many centres [4, 7, 10, 19].

MPT provides the most lateral perspective to the Sylvian fissure and carotid cistern among the three craniotomies analysed here. A focal (≤ 2 cm) opening of the fissure at the point between the vertical and horizontal segments provides a sufficient window on the MCA bifurcation [7, 9]. Yet, when targeting ICA bifurcation or PCom, dissection needs to be extended deeper on the vertical part of the Sylvian fissure and towards the carotid and crural cisterns.

This lateral-to-medial (distal-to-proximal) dissection cannot be obtained through the TO corridor, given its purely ventral projection. In the latter, the focus split is centred at the M1 segment, and a proximal-to-distal dissection is performed to expose the MCA bifurcation if needed. Proximal-to-distal dissection of the M1 segment may become difficult through the MTP approach if interfascial dissection and complete drilling of the sphenoid wing is not performed [28]. However, these steps may cause potential damage to the facial nerve, temporalis muscle atrophy, and bony cosmetic defects, most of which can be spared with the TO approach.

### Keyhole approach selection for each vascular target

As previously stressed, approach selection will predominantly rely on surgeon preference and experience; yet objective and comparative data can help guiding decision making, particularly in borderline cases or with the advent of new routes, such as the TO for cerebrovascular procedures [17, 24].

Freedom of instrument handling is determined by superficial exposure area rather than by the surgical corridor volume. This measurement (surgical freedom) is considered a surrogate of manoeuvrability and, when applied to specific vascular segments, may become a limiting factor for achieving adequate dissection and vessel exposure [16]. Complimentarily, the range of instrument angulation when targeting a specific vascular segment may be determinant for the application of a vascular clip and could give an idea of favourable and unfavourable aneurysm projections for each keyhole approach [22].

Following our data, the TO offers the highest range of motion at the M1, followed by the MCA bifurcation; while access to more medial structures (i.e., PCom and ICA) is more constraint even when lateral orbital rim has been removed. Instrument angulation with the TO is also maximal at the M1. Noteworthy, at the MCA bifurcation ventral angulation with TO is rather limited, which may difficult clip placement for superiorly or inferiorly projecting aneurysms. In our illustrative case, the TO was selected for a laterally projecting MCA aneurysm with a rather short M1 segment. An important point to consider is the restrictions of the TO corridor; even when these are somewhat circumvented by transitory removal or the lateral orbital rim, the application of multiple clips may hamper visualization of the field. Moreover, the TO corridor is more suitable for fully endoscopic or endoscopically assisted techniques, which may offer some limitations to arachnoid dissection particularly with a swollen brain or active hemorrhage. For these reasons, we consider the TO route for elective cases and would refrain from indicating this route in acute subarachnoid hemorrhage. Another critical step is preoperative planning and selection of the adequate case in terms of anatomical location and dome projection. Besides, the TO craniectomy and final reconstruction is rather standardized and straight forward technique under experienced hands.

LSO approach offers the greatest freedom of movement at the ICA bifurcation and M1. Angulation is also maximal at these segments but remains rather high also at the PCom and MCA bifurcation. MPT craniotomy provided the highest range of movement at all four vascular segments compared to LSO and TO approaches. Based on our data, MPT seems particularly suitable for MCA bifurcation and PCom aneurysms, providing not only the highest surgical freedom at these targets, but also the greatest degree of ventral and horizontal angulation. These results are in line with previous reports [20]. Given its versatility, in our illustrative case MPT was chosen for a case of double aneurysm clipping (MCA and ICA bifurcation).

Esposito et al. proposed a cut-off distance of 15 mm between the M1 origin and the aneurysmal neck as an objective criterion to choose between supraorbital and MPT craniotomy for MCA aneurysms [10]. Other clinical and anatomical analysis have shown that favourable results can also be obtained with either of the two keyhole approaches [4, 20]. Generally speaking, and supported by our data, MPT may be preferred for cases involving a PCom aneurysm or when there is a long M1 in MCA aneurysms; meanwhile, LSO may be chosen for ICA bifurcation aneurysms or MCA aneurysms when there is a short M1 [3, 4].

TO approach deserves further clinical evaluation, but as for now, it seems particularly suitable for MCA aneurysms when there is a short M1 segment. This may seem superimposed to the LSO indications, however, the ventral perspective of the TO and the distinct Sylvian fissure splitting technique may reduce manipulation of the frontal operculum, which may come as a potential advantage compared to the LSO [14].

### Limitations

The concept of surgical manoeuvrability is aimed at representing the maximal range of movement achievable with microsurgical instruments at a specific surgical target and through a specific surgical route. In the present study, manoeuvrability is calculated based on Heron's formula, and represented by an area of surgical freedom and a degree of ventral and horizontal angulations, however, other methods have been described [15, 16]. Modelling surgical manoeuvrability is an unprecise estimation, particularly in fixated cadaver specimens, in which tissue elastance and brain compliance significantly differ from that of the living human. Therefore, extrapolation of data and conclusions to the surgical scenario must be done with caution.

## Conclusions

An objective anatomical analysis comparing three keyhole approaches for ICA and MCA aneurysms is provided by quantifying morphometric parameters for specific vascular targets. The ventral TO route offers comparable manoeuvrability to M1 and MCA bifurcation than the ventro-lateral LSO and the lateral MPT craniotomies. However, TO features a narrower surgical corridor to mesial vascular targets, such as ICA bifurcation and PCom. MPT and LSO seem comparable to target all vascular segments hereby analysed.

## Supplementary Information

Below is the link to the electronic supplementary material.

Supplementary file1 (DOCX 27 KB)

## Data Availability

No datasets were generated or analysed during the current study.
