# Supplementary material for: Selecting the optimal keyhole approach for internal carotid and middle cerebral artery aneurysms. Anatomical comparison of transorbital, lateral supraorbital and minipterional routes with clinical implications
Source: Acta Neurochir (Wien). 2026 Apr 13;168(1):107. doi: 10.1007/s00701-026-06871-x (PMC13183721; doi:10.1007/s00701-026-06871-x)
Supplement: Supplementary file 1 — Supplementary file1 (DOCX 27 KB) [file 701_2026_6871_MOESM1_ESM.docx]

SUPPLEMENTARY TABLES

Supplementary Table S1. Post hoc analysis detailing the mean differences in the manoeuvrability measurements when targeting the PCom with the three keyhole approaches: transorbital (TO), lateral supraorbital (LSO) and minipterional (MTP). Data are given as mean differences (SD).

| **PCOM** | | | | |
| --- | --- | --- | --- | --- |
| **Manoeuvrability variable** | **Reference approach** | **Comparison approach** | **Differential** | **p-value** |
| Surgical freedom area (cm^2^) | TO | MPT | -93.40 (28.72) | **0.030** |
|  |  | LSO | -37.37 (28.72) | 0.677 |
|  | MPT | TO | 93.40 (28.72) | **0.030** |
|  |  | LSO | 56.03 (33.17) | 0.376 |
|  | LSO | TO | 37.37 (28.72) | 0.677 |
|  |  | MPT | -56.03 (33.17) | 0.376 |
| Ventral angulation (º) | TO | MPT | -14.47 (8.09) | 0.322 |
|  |  | LSO | -8.59 (8.09) | 0.948 |
|  | MPT | TO | 14.47 (8.09) | 0.322 |
|  |  | LSO | 5.87 (9.34) | 1.000 |
|  | LSO | TO | 8.59 (8.09) | 0.948 |
|  |  | MPT | -5.87 (9.34) | 1.000 |
| Horizontal angulation (º) | TO | MPT | -17.99 (2.87) | **< 0.001** |
|  |  | LSO | -8.09 (2.87) | **0.060** |
|  | MPT | TO | 17.99 (2.87) | **< 0.001** |
|  |  | LSO | 9.91 (3.31) | **0.046** |
|  | LSO | TO | 8.09 (2.87) | **0.060** |
|  |  | MPT | -9.91 (3.31) | **0.046** |

Supplementary Table S2. Post hoc analysis detailing the mean differences in the manoeuvrability measurements when targeting the ICA bifurcation with the three keyhole approaches: transorbital (TO), lateral supraorbital (LSO) and minipterional (MTP). Data are given as mean differences (SD).

| **ICA BIF** | | | | |
| --- | --- | --- | --- | --- |
| **Manoeuvrability variable** | **Reference approach** | **Comparison approach** | **Differential** | **p-value** |
| Surgical freedom area (cm^2^) | TO | MPT | -55.06 (15.42) | **0.018** |
|  |  | LSO | -58.58 (15.42) | **0.013** |
|  | MPT | TO | 55.06 (15.42) | **0.018** |
|  |  | LSO | -3.52 (17.81) | 1.000 |
|  | LSO | TO | 58.58 (15.42) | **0.013** |
|  |  | MPT | 3.52 (17.81) | 1.000 |
| Ventral angulation (º) | TO | MPT | -14.85 (3.41) | **0.006** |
|  |  | LSO | -16.34 (3.41) | **0.003** |
|  | MPT | TO | 14.85 (3.41) | **0.006** |
|  |  | LSO | -1.49 (3.94) | 1.000 |
|  | LSO | TO | 16.34 (3.41) | **0.003** |
|  |  | MPT | 1.49 (3.94) | 1.000 |
| Horizontal angulation (º) | TO | MPT | -8.06 (3.53) | 0.145 |
|  |  | LSO | -7.91 (3.53) | 0.156 |
|  | MPT | TO | 8.06 (3.53) | 0.145 |
|  |  | LSO | 0.16 (4.08) | 1.000 |
|  | LSO | TO | 7.91 (3.53) | 0.156 |
|  |  | MPT | -0.16 (4.08) | 1.000 |

Supplementary Table S3. Post hoc analysis detailing the mean differences in the manoeuvrability measurements when targeting the M1 segment with the three keyhole approaches: transorbital (TO), lateral supraorbital (LSO) and minipterional (MTP). Data are given as mean differences (SD).

| **M1** | | | | |
| --- | --- | --- | --- | --- |
| **Manoeuvrability variable** | **Reference approach** | **Comparison approach** | **Differential** | **p-value** |
| Surgical freedom area (cm^2^) | TO | MPT | -23.53 (24.75) | 1.000 |
|  |  | LSO | -31.32 (24.75) | 0.713 |
|  | MPT | TO | 23.53 (24.75) | 1.000 |
|  |  | LSO | -7.79 (28.59) | 1.000 |
|  | LSO | TO | 31.32 (24.75) | 0.713 |
|  |  | MPT | 7.79 (28.59) | 1.000 |
| Ventral angulation (º) | TO | MPT | 1.00 (14.04) | 1.000 |
|  |  | LSO | 2.92 (14.04) | 1.000 |
|  | MPT | TO | -1.00 (14.04) | 1.000 |
|  |  | LSO | 1.92 (16.22) | 1.000 |
|  | LSO | TO | -2.92 (14.04) | 1.000 |
|  |  | MPT | -1.92 (16.22) | 1.000 |
| Horizontal angulation (º) | TO | MPT | 3.66 (13.97) | 1.000 |
|  |  | LSO | -1.19 (13.97) | 1.000 |
|  | MPT | TO | -3.66 (13.97) | 1.000 |
|  |  | LSO | -4.86 (16.14) | 1.000 |
|  | LSO | TO | 1.19 (13.97) | 1.000 |
|  |  | MPT | 4.86 (16.14) | 1.000 |

Supplementary Table S4. Post hoc analysis detailing the mean differences in the manoeuvrability measurements when targeting the MCA bifurcation with the three keyhole approaches: transorbital (TO), lateral supraorbital (LSO) and minipterional (MTP). Data are given as mean differences (SD).

| **MCA BIF** | | | | |
| --- | --- | --- | --- | --- |
| **Manoeuvrability variable** | **Reference approach** | **Comparison approach** | **Differential** | **p-value** |
| Surgical freedom area (cm^2^) | TO | MPT | -109.37 (52.01) | 0.206 |
|  |  | LSO | -29.63 (60.01) | 1.000 |
|  | MPT | TO | 109.37 (52.01) | 0.206 |
|  |  | LSO | 79.73 (67.15) | 0.807 |
|  | LSO | TO | 29.63 (60.01) | 1.000 |
|  |  | MPT | -79.73 (67.15) | 0.807 |
| Ventral angulation (º) | TO | MPT | -28.36 (8.18) | **0.026** |
|  |  | LSO | -12.78 (9.54) | 0.641 |
|  | MPT | TO | 28.36 (8.18) | **0.026** |
|  |  | LSO | 15.59 (10.57) | 0.536 |
|  | LSO | TO | 12.78 (9.54) | 0.641 |
|  |  | MPT | -15.59 (10.57) | 0.536 |
| Horizontal angulation (º) | TO | MPT | -6.63 (10.53) | 1.000 |
|  |  | LSO | -2.81 (12.16) | 1.000 |
|  | MPT | TO | 6.63 (10.53) | 1.000 |
|  |  | LSO | 3.82 (13.59) | 1.000 |
|  | LSO | TO | 2.81 (12.16) | 1.000 |
|  |  | MPT | -3.82 (13.59) | 1.000 |
